# Hand Hygiene Practices Among Indian Medical Undergraduates: A Questionnaire-Based Survey

**DOI:** 10.7759/cureus.1463

**Published:** 2017-07-12

**Authors:** Pranav D Modi, Pooja Kumar, Rajavi Solanki, Janhavi Modi, Srinath Chandramani, Niharika Gill

**Affiliations:** 1 Trainee Research Assistant, Clinsearch Healthcare Solutions, Thane, Maharashtra; Dept. of Internal Medicine, K.J. Somaiya Hospital and Medical Research Centre, Mumbai; 2 MGM Univeristy of Health Sciences, MGM Medical College, Navi Mumbai, India; 3 Intern, DY Patil Medical University; 4 Terna College, Maharashtra University of Health Sciences; 5 Internal Medicine, Geriatric Medicine, MBBS, Dnb (medicine) Pgdgm ( Post Graduate Diploma In Geriatric Medicine) Associate Professor K.j. Somaiya Hospital and Medical Research Centre, Mumbai; 6 Internal Medicine, Rheumatology, Professor and Head, Dept of Medicine, K.j. Somaiya Hospital and Medical Research Centre, Mumbai

**Keywords:** hand hygiene, my 5 moments, hand washing, hand rubbing, who, cdc, world hand hygiene day, global handwashing day

## Abstract

Background and objectives

To prevent the spread of infections in all healthcare settings, hand hygiene must be routinely practiced. Appropriate hand hygiene techniques can go a long way in reducing nosocomial infections, cross-transmission of microorganisms and the risk of occupational exposure to infectious diseases. World Health Organisation (WHO) has taken an incredible approach called “My Five Moments for Hand Hygiene" which defines the key moments when health-care workers should perform hand hygiene. We thus carried out a survey to assess knowledge of hand hygiene practices among undergraduate medical students.

Materials and methods

A cross-sectional survey was conducted among 523 Indian medical undergraduates. The questionnaire used was adapted from the WHO hand hygiene knowledge questionnaire for health-care workers and was distributed both, in print and online formats. The response to each question was examined using percentages.

Results

Nearly 57% (n=298) of medical students who participated in this study did not receive any formal training in hand hygiene. Only 27% (n=141) students knew that the most frequent source of germs responsible for health-care associated infections were the germs already present on or within the patient. Nearly 68.6% (n= 359) students were unaware of the sequence of hand washing and hand rubbing. Although 71.9% (n=376 ) students claimed that they use an alcohol-based hand rub routinely, only 36.1% (n=189 ) students knew the time required for a hand rub to kill the germs on the hands. Overall hand hygiene knowledge was low in 6.9% (n=36), moderate in 80.9% (n=423) and good in 12.2% (n=23) of respondents.

Conclusions

The awareness about hand hygiene practices among medical students is low. Nearly 57% (n=298) of the respondents never received any formal training in hand hygiene throughout their course of medical undergraduate study. To prevent the spread of infections in healthcare settings, medical students should be given proper training in hand hygiene practices right from the first year of the medical curriculum. This should be done by running workshops and annual seminars on hand hygiene practices and making it a requisite for clinical skills assessment.

## Introduction

Infections acquired while receiving health care have significantly increased the mortality rate around the world by thousands. Hands have been identified as the major source of germ transmission while providing health care [1]. Every person involved in patient care, healthcare worker or otherwise, should be able to accurately perform hand hygiene and at the appropriate time. World Health Organisation (WHO) has defined certain guidelines on how to perform hand hygiene. The alcohol-based formulation is preferred for routine hygienic disinfection if hands are not visibly dirty. Compared to handwashing with soap and water, it is not only more rapid and effectual but also better tolerated by hands. When hands are noticeably soiled, contaminated by blood or other bodily fluids, it is recommended to use soap and water. In the case of exposure to spore-forming pathogens, including Clostridium difficile, handwashing with soap and water is strongly advised [1].

Precise use of hand hygiene techniques can go a long way in reducing cross-transmission of microorganisms, nosocomial infections and the risk of occupational exposure to infectious diseases. Klebsiella spp, Staphylococcus aureus, Clostridium difficile, Methicillin-resistant Staphylococcus aureus (MRSA) and gram-negative bacteria are some of the organisms that are likely to be found on healthcare workers’ hands. However, direct patient contact is not the sole method of pathogen transmission. Bacteria can also be acquired on the workers’ hands by touching contaminated surfaces in the patient environment [2].

A study done in a rural Indian hospital showed that the proportion of healthcare workers that reported to ‘always’ practicing hand hygiene in the selected situations varied from 40–96% [3]. In 2014, results of a project known as the Healthcare-Associated Infections (HAI) Prevalence Survey described the burden of healthcare-associated infections (HAI) in the United States hospitals and reported that, in 2011, there were an estimated 722,000 HAIs in U.S. acute care hospitals. Additionally, about 75,000 patients with HAIs died during their hospitalizations. More than half of all HAIs occurred outside of the intensive care unit [4]. The result of a study conducted by Post Graduate Institute of Medical Education and Research (PGIMER), New Delhi in 2014, had an overall healthcare-associated infections (HAI) prevalence of 8.78% with highest in intensive care unit (ICUs) (33.3%) followed by pediatric wards (12.5%) and surgical wards (10.3%) [5]. In 2015, the Indian pooled mean HAI rates for all infections were above The Centers for Disease Control and Prevention/National Healthcare Safety Network (CDC/NHSN) percentile threshold [6]. Studies conducted in various countries also show a high prevalence of healthcare associated infections.

In Pakistan, a study conducted in 2009 revealed that only 4.7% of the physicians decontaminated their hands before having direct contact with their patients. Only 17% claimed to be aware of the WHO recommendations on hand hygiene. The majority of subjects considered the lack of sinks, soap, water and disposable towel as a major barrier towards hand hygiene adherence. Overall compliance of hand hygiene was found to be 38.8% but it widely varied as a function of patient care activity [7]. Another similar survey was conducted in 2015, in Gulbarg, Karnataka, which was based on the hand hygiene knowledge questionnaire for health-care workers by the WHO. The study showed overall moderate knowledge among the students [8].

A study carried out in a group of 414 students of Jagiellonian University Medical College in Poland revealed that professional practice of 22.9% of students was not preceded by any training in the field of hospital hygiene and in 28% of cases, training prior to internship did not cover hand hygiene [9]. A study to assess hand hygiene knowledge and practices among health-care workers in a teaching hospital in Ghana showed knowledge in hand hygiene practices to be fair. Heavy patient load, forgetfulness, and unavailability of water and detergent were major contributing factors hampering proper hand hygiene practices. Also, there was low patronage for alcohol-based hand rubs and only 5.3% had access to warm running water [10]. During 2013, New Zealand national data showed poor compliance with hand hygiene practices by medical students [11]. Therefore we conducted a survey in an attempt to assess the existing knowledge regarding hand hygiene practices among Indian medical undergraduates.

## Materials and methods

This was a questionnaire-based cross-sectional survey to assess the knowledge of hand hygiene practice among the undergraduate medical students. This study was conducted in April-May 2017 and the study documents were reviewed and approved by the Institutional Ethics Committee (IEC) of K. J. Somaiya Medical College and Hospital, Mumbai, Maharashtra. All participants had to complete a 25-item self-administered WHO hand hygiene questionnaire for health-care workers [12]. The questionnaire was filled by the respondents in either print or electronic format. Consent was obtained from all participants and participation was voluntary. Convenient sampling method was used for data collection and medical undergraduates from the first year up to the intern year were included in the study from various medical colleges across India. Data were analyzed using percentages. Correct answers were given one point whereas incorrect answers scored zero. The maximum score achievable for knowledge was 25 points. The level of hand hygiene knowledge was calculated by dividing the responses into three groups based on a score of more than 75% considered as good, 50-74% moderate, and less than 50% considered as low.

## Results

The responses of participants to the survey based on hand hygiene knowledge questionnaire for health-care workers are provided in Table 1.

**Table 1:** Table representing the responses to survey based on hand hygiene knowledge questionnaire for health-care workers by World Health Organisation (WHO)

Sr No.	Question	Correct Answer	% Population Answered (Correctly)	% Population Answered (Incorrectly)
1.	Did you receive formal training in hand hygiene in the last three years?	-	43% Said Yes (n=225)	57% Said No (n = 298)
2.	Do you routinely use an alcohol-based handrub for hand hygiene?	-	71.9% Said Yes (n=376)	28.1% Said No (n = 147)
3.	Which of the following is the main route of cross-transmission of potentially harmful germs between patients in a health-care facility?	Health-care workers’ hands when not clean	50.7% (n = 265)	49.3% (n = 258)
4.	What is the most frequent source of germs responsible for health care-associated infections?	Germs already present on or within the patient	27% (n = 141)	73% (n = 382)
5.	Which of the following hand hygiene actions prevents transmission of germs to the patient?			
	a. Before touching a patient	Yes	94.3% (n = 493)	5.7% (n = 30)
	b. Immediately after a risk of body fluid exposure	Yes	86.6% (n = 453)	13.4% (n = 70)
	c. After exposure to the immediate surroundings of the patient	No	24.3% (n = 127)	75.7% (n = 396)
	d. Immediately before a clean/aseptic procedure	Yes	90.8% (n = 475)	9.2% (n = 48)
6.	Which of the following hand hygiene actions prevents transmission of germs to the health-care worker?			
	a. After touching a patient	Yes	91.4% (n = 478)	8.6% (n = 45)
	b. Immediately after a risk of body fluid exposure	Yes	90.2% (n = 472)	9.8% (n = 51)
	c. Immediately before a clean/aseptic procedure	No	22.6% (n = 118)	77.4% (n = 405)
	d. After exposure to immediate surroundings of the patient	Yes	82.2% (n = 430)	17.8% (n = 93)
7.	Which of the following statements on alcohol-based handrub and handwashing with soap and water are true?			
	a. Handrubbing is more rapid for hand cleansing than handwashing	True	61.4% (n=321)	38.6% (n = 202)
	b. Handrubbing causes skin dryness more than handwashing	False	57.7% (n = 302)	42.3% (n = 221)
	c. Handrubbing is more effective against germs than handwashing	False	68.1% (n = 356)	31.9% (n = 167)
	d. Handwashing and handrubbing are recommended to be performed in sequence	False	31.4% (n = 164)	68.6% (n = 359)
8.	What is the minimal time needed for alcohol-based handrub to kill most germs on your hands?	20 seconds	36.1% (n = 189)	63.9% (n = 334)
9.	Which type of hand hygiene method is required in the following situations?			
	a. Before palpation of the abdomen	Rubbing	67.1% (n = 351)	32.9% (n = 172)
	b. Before giving an injection	Rubbing	43.4% (n = 227)	56.6% (n = 296)
	c. After emptying a bedpan	Washing	80.1% (n = 419)	19.9% (n = 104)
	d. After removing examination gloves	Washing	76.7% (n = 401)	23.3% (n =122)
	e. After making a patients bed	Rubbing	27.9% (n = 146)	72.1% (n = 377)
	f. After visible exposure to blood	Washing	82.3% (n = 430)	17.7% (n = 93)
10.	Which of the following should be avoided, as associated with increased likelihood of colonisation of hands with harmful germs?			
	a. Wearing jewellery	Yes	68.5% (n = 358)	31.5% (n=165)
	b. Damaged skin	Yes	94.3% (n = 493)	5.7% (n=30)
	c. Artificial fingernails	Yes	88.3% (n = 462)	11.7% (n=61)
	d. Regular use of a hand cream	No	59.5% (n=311)	40.5% (n=212)

**Figure 1:**
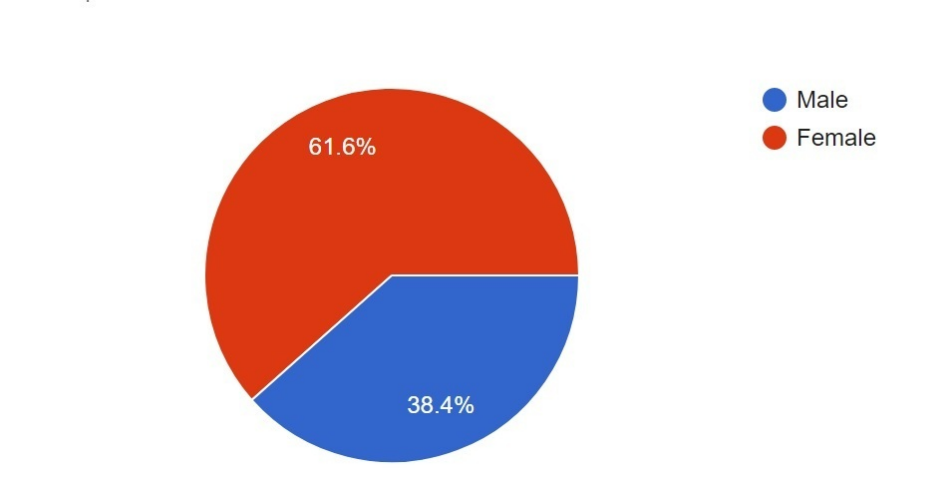
Distribution of participants according to sex

Out of 1000 medical students that we approached, 523 responded with a response rate of 52.3% (n=523). Nearly, 61.1% (n=322) of the respondents were females and 38.4% (n=201) were males (Figure 1).

The recorded percentage of responders from Mumbai was 85% (n = 444), Navi Mumbai was 7.1% (n=37) and rest of India was 7.9% (n=42). The respondents were 21.1% (n=110), 14.8% (n=77), 14.2% (n=74), 14.3% (n=30), 39% (n=204) from the first year, second year, third year, fourth year and intern year of medical school, respectively.

**Figure 2:**
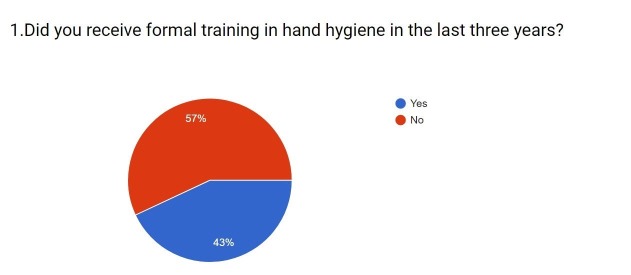
Responses to question one

It was observed that only 43% (n=225) of the medical students have received formal training in hand hygiene during the course of their medical graduation (Figure 2). Nearly 57% (n=298) students did not receive any formal training in hand hygiene. However, 71.9% (n=376) use an alcohol-based hand rub daily.

When asked about the main route of cross-transmission of potentially harmful germs between patients in a health-care facility, only 50.7% (n=265) agreed that it’s the worker’s hands were not clean. The rest answered that it’s the patient’s exposure to colonised surfaces (beds, chairs, tables, floor), air circulating in the hospital, sharing non-invasive objects between patients (stethoscope, pressure cuffs, etc) in a number of 29.6% (n=155 ), 11.9% (n=62), 7.8% (n=41 ), respectively.

**Figure 3:**
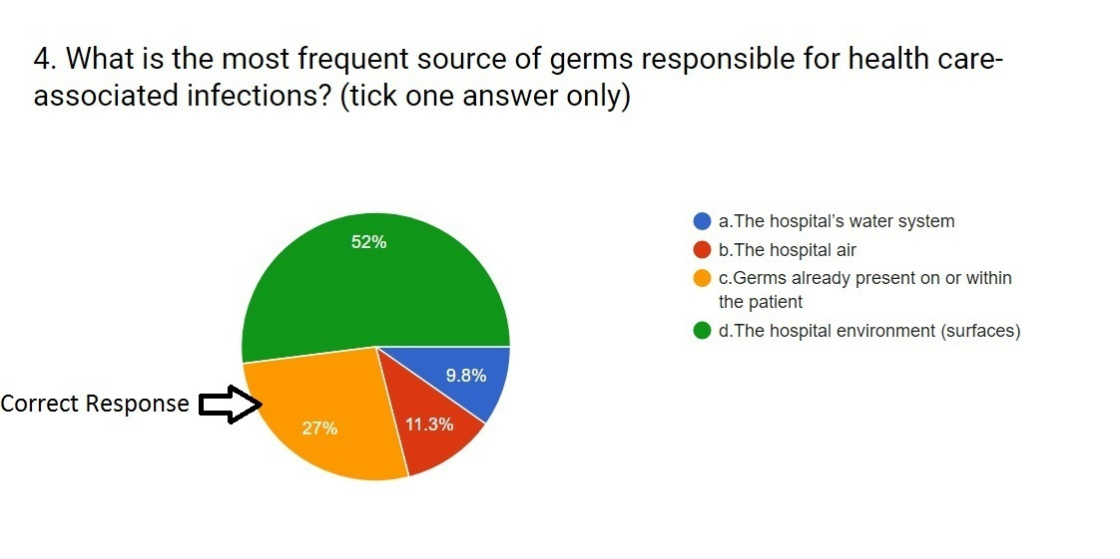
Responses to question four

When asked about the most frequent source of germs responsible for health care-associated infections, only 27% (n=141 ) knew that it was the germs that are already present on or within the patient. More than half of them thought that the hospital environment was the main source. A few of them thought it was the hospital air that was the source of infection (11.3%; n=59), and 9.8% (n=51) thought that the hospital’s water system was the main source of infection. A majority of 52% (n=272) thinks that it is the hospital environment (Figure 3).

Out of the five moments of hand hygiene, better awareness was observed regarding actions like hand hygiene before touching a patient (94.3%; n=493), immediately before a clean/aseptic procedure (90.8%; n=475), immediately after the risk of body fluid exposure (86.6%; n=453). Though only 24.3% (n=127) population knew that hand hygiene after exposure to immediate surroundings of the patient does not prevent transmission of germs to the patient.

Better awareness was recorded regarding actions like hand hygiene after touching a patient (91.4%; n=478), hand hygiene immediately after the risk of body fluid exposure (90.2%; n=472), and hand hygiene after exposure to immediate surroundings of the patient (82.2%; n=430). Only 22.6% (n=118) people knew that hand hygiene before a clean/aseptic procedure does not prevent transmission of germs to the health care worker.

When knowledge about handwashing with soap and water and hand rubbing with an alcohol-based hand rub was tested, 61.4% (n=321) people agreed that hand rubbing is more rapid for hand cleansing than hand washing. Nearly 57.7% (n=302) people agreed that hand rubbing does not cause skin dryness more than hand washing and 68.1% (n=356) population knew that hand rubbing is not more effective against germs than hand washing. And only 31.4% (n=164) were aware that hand washing and hand rubbing are not recommended to be performed in that sequence.

**Figure 4:**
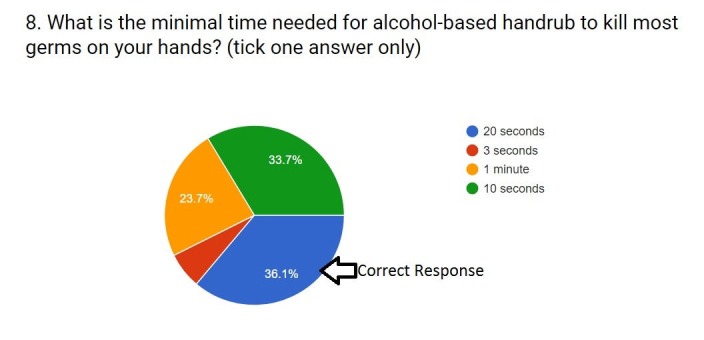
Responses to question eight

Only 36.1% (n=189) people knew that the minimum time required by alcohol-based hand rub to kill germs on hands is 20 seconds. And 63.9% (n=334) of the participants did not know the minimum time required by the alcohol-based hand rub to kill the germs on hands (Figure 4).

When asked about the hygiene methods required in given situations, people agreed that hand rubbing is required before palpation of the abdomen (67.1%; n=351), before giving an injection (43.4%; n=227) and after making a patient’s bed (27.9%; n=146). Students were also aware that hand washing was necessary after emptying a bedpan (80.1%; n=419), after removing examination gloves (76.7%; n=401) and after visible exposure to blood (82.3%; n=430).

These medical students agreed that wearing jewelry (68.5%; n=358), damaged skin (94.3%; n=493) and artificial fingernails (88.3%; n=462) are associated with increased risk of colonization of hands with harmful germs. And 59.5% (n=311) knew that regular use of a hand cream did not increase the risk of colonization of hands with germs.

The overall scores for the level of hand hygiene knowledge is provided in Table 2.

**Table 2:** Table representing the level of hand hygiene knowledge Low: less than 50% (score below 12.5) Moderate: 50-74% (score between 12.5 to 18.5) Good: 75% & above (score of 18.75 and above)

Level of hand hygiene knowledge	Low	Moderate	Good
Percentage	6.9%	80.9%	12.2%
Number of respondents (n)	36	423	64

## Discussion

**Figure 5:**
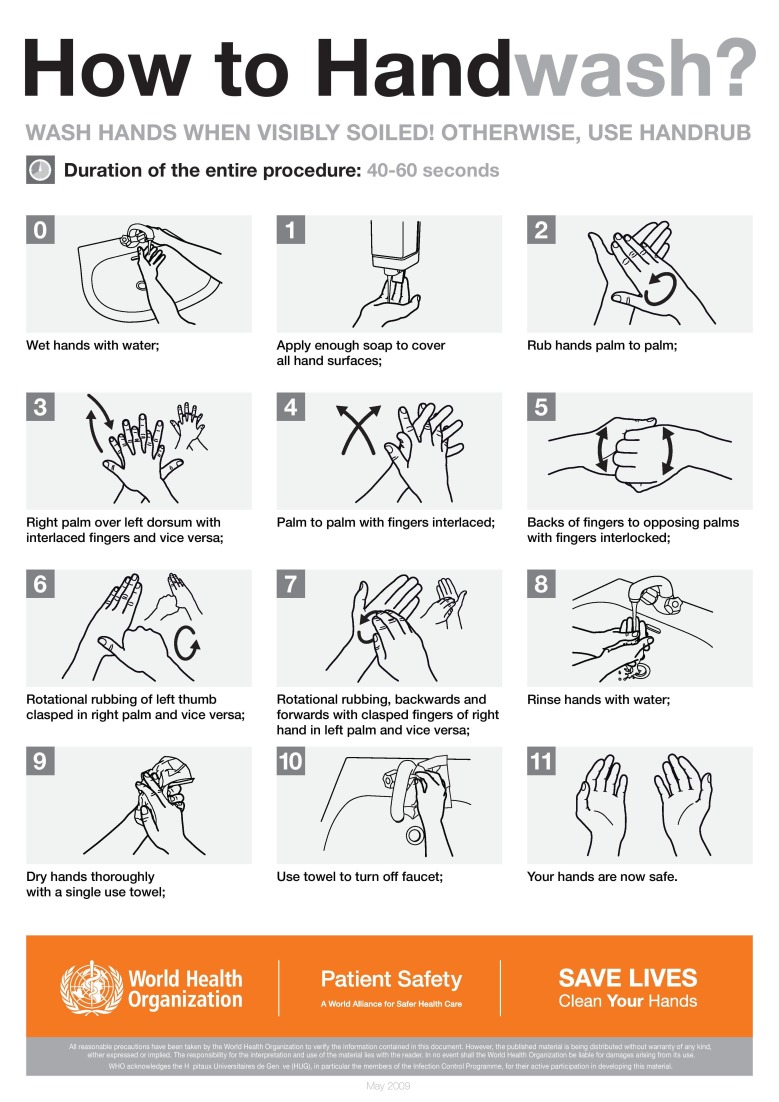
Hand washing technique by World Health Organisation (WHO)

**Figure 6:**
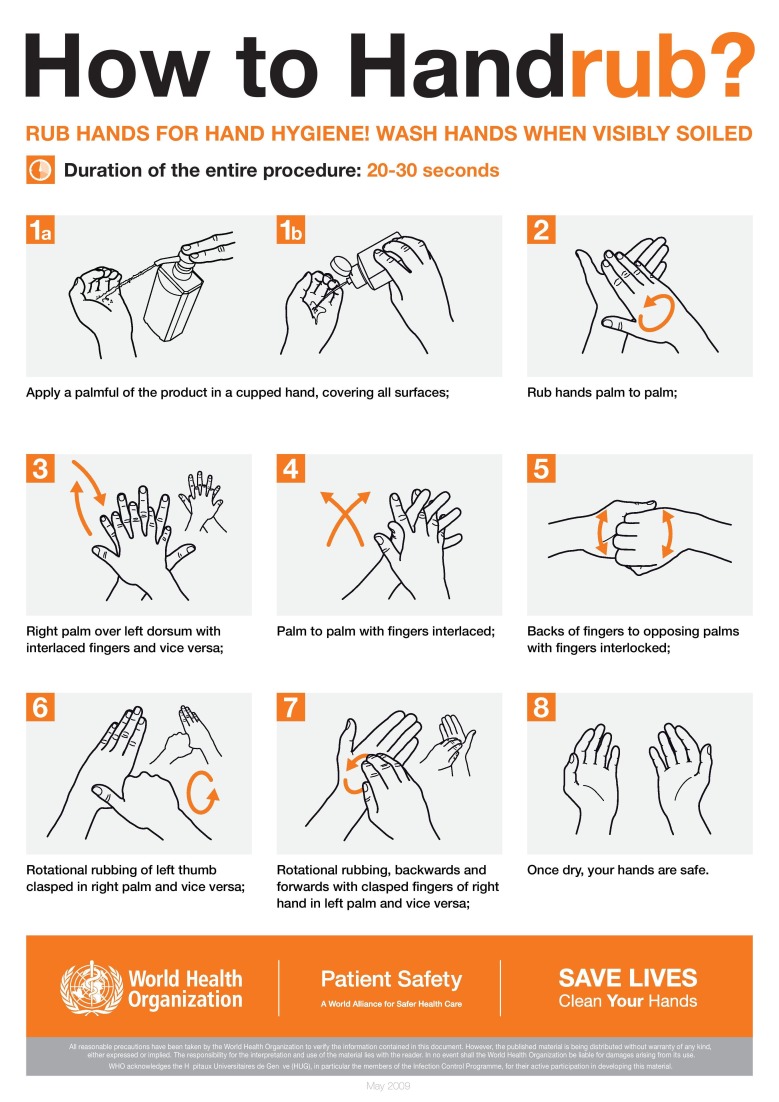
Hand rubbing technique by World Health Organisation (WHO)

Hands can be cleaned by two methods, namely handwashing (Figure 5) and hand rubbing (Figure 6). Hand washing is carried out with soap and water whereas hand rubbing is completed with an alcohol-based hand rub. Hands should be washed for at least 15 seconds in order to kill the germs while making sure that all areas of the hands are cleaned properly. In a healthcare setting, hand rubbing is the preferred method for cleaning hands. It takes less time, it kills the potentially deadly germs more effectively than a soap does, it does not dry or irritate the skin unlike soap and is more accessible than a hand washing sink.

While cleaning hands with a hand rub, care should be taken that areas between fingers, thumb, and fingertips are not missed. Also, it should be noted that hand cleaning is required even after removing gloves. The effectiveness of a hand rub depends on the quantity of the alcohol-based sanitizer used for cleaning. The more the quantity, the better the cleaning. Clostridium difficile is a very common infection in a healthcare setting that causes severe diarrhea. Its spores cannot be killed by an alcohol-based sanitizer. So this infection can only be prevented by wearing gloves before examining a patient that is infected and washing hands with soap and water after the examination is done.

**Figure 7:**
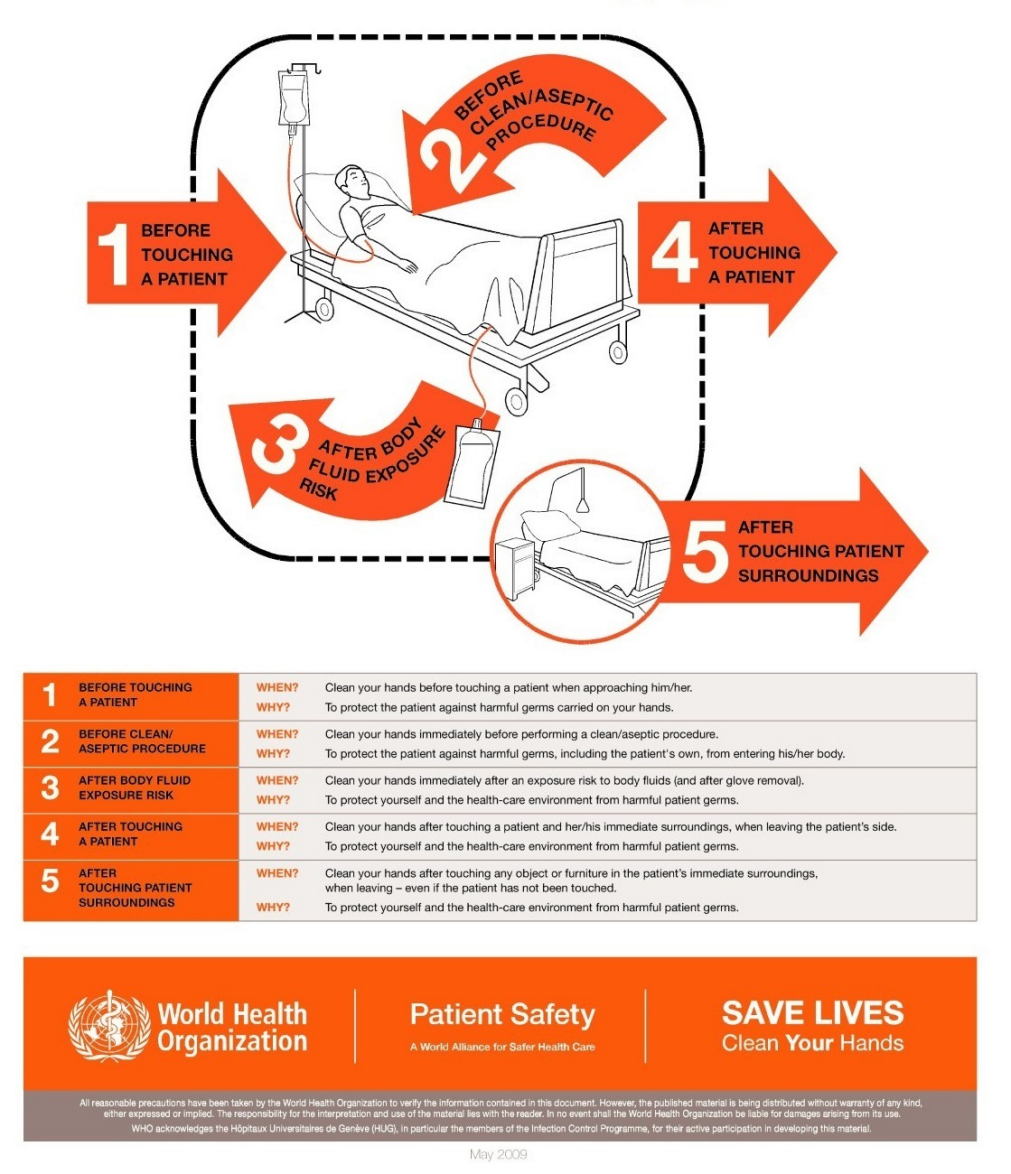
Five moments of hand hygiene by World Health Organisation (WHO)

To get rid of the infections caused by lack of hand hygiene, the WHO designed the ‘My Five moments of hand hygiene’ in a very simplified manner that helped the health care workers to understand, train, monitor and report hand hygiene more effectively in day to day practice. As per this design (Figure 7), the health-care workers should clean their hands before touching a patient, before clean/aseptic procedures, after body fluid exposure/risk, after touching a patient and after touching patient surroundings [13].

**Figure 8:**
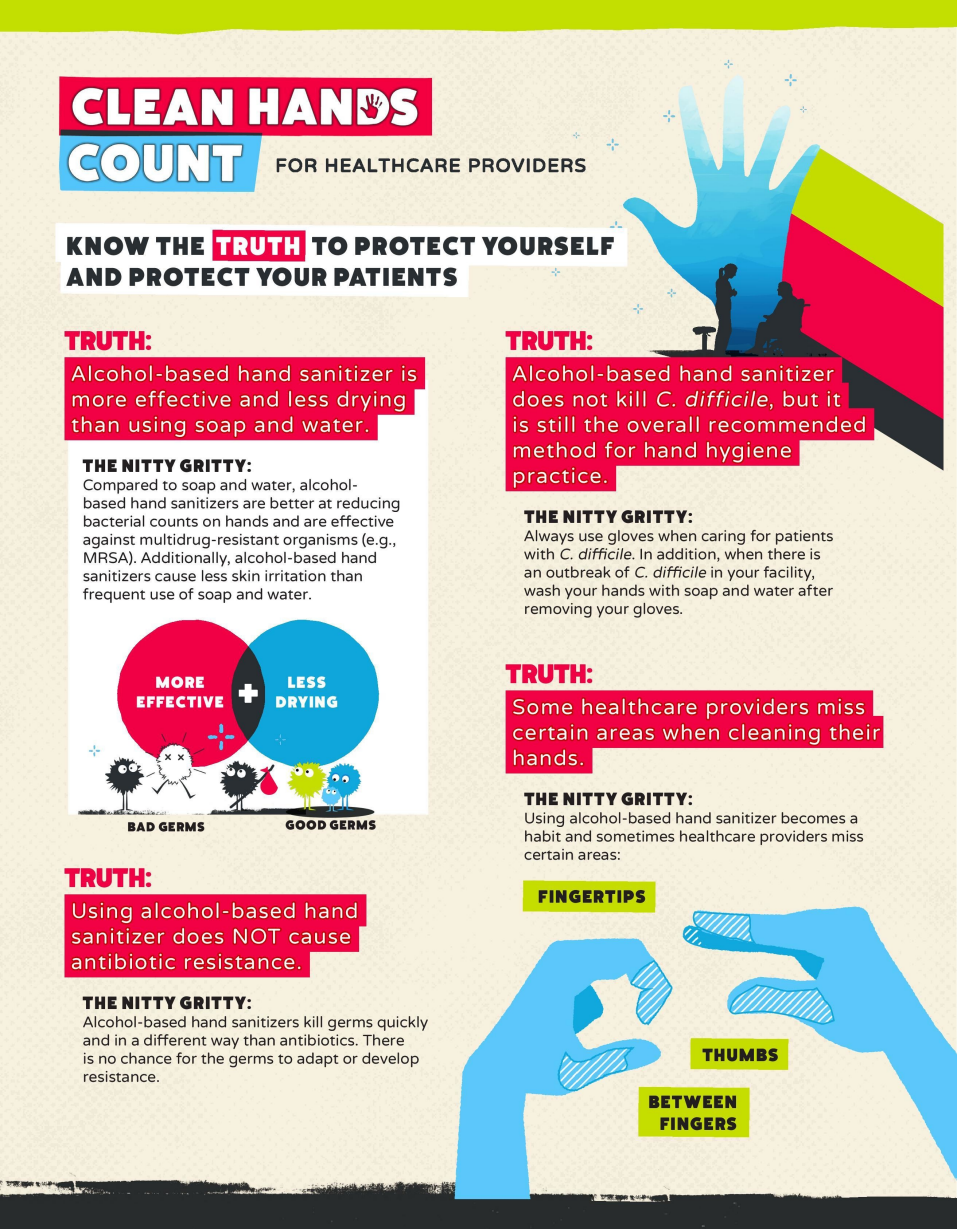
The Centers for Disease Control and Prevention (CDC's) "Clean Hands Count" campaign: Health-care providers fact sheet This material was developed by The Centers for Disease Control and Prevention (CDC).(www.cdc.gov/HandHygiene).

**Figure 9:**
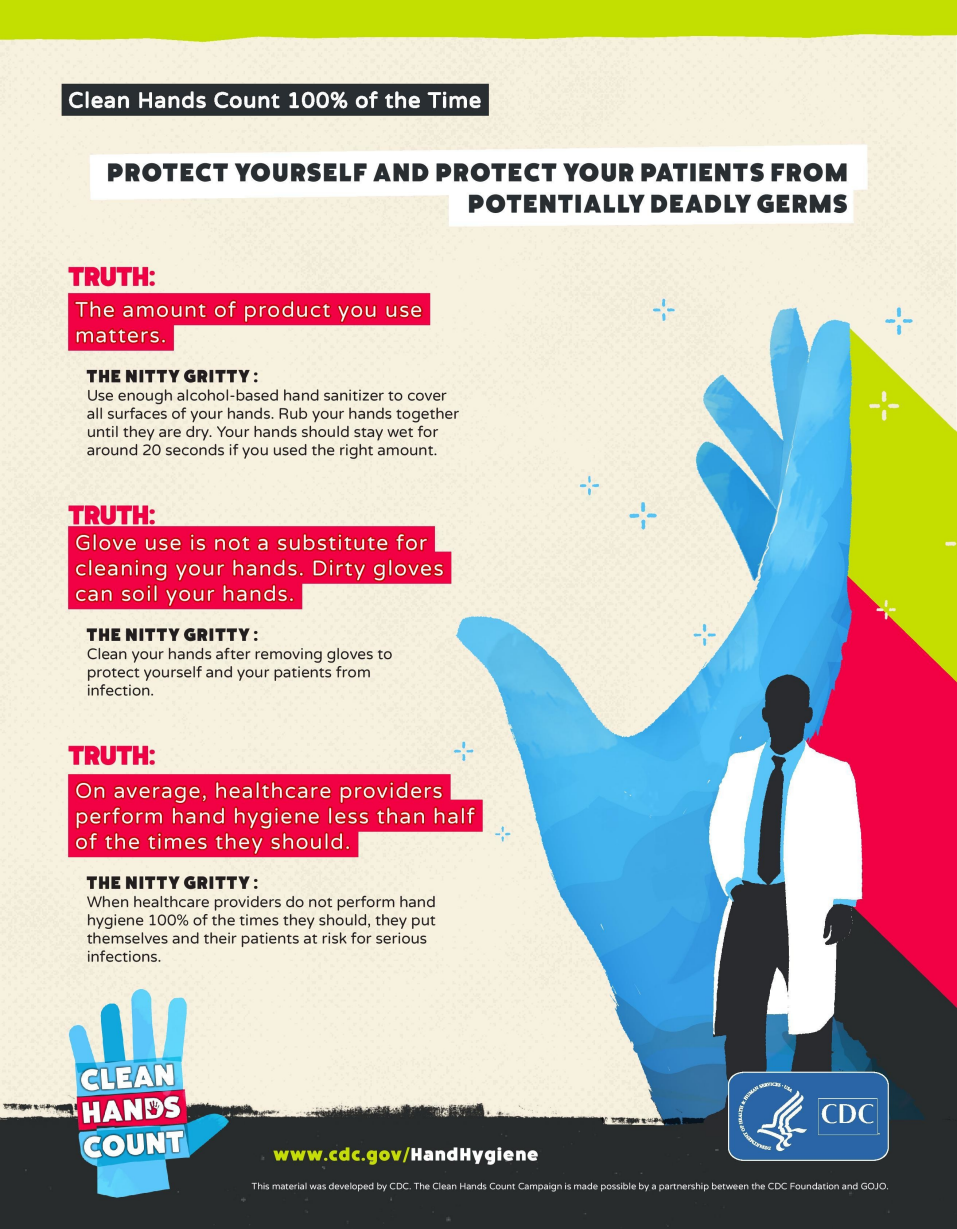
The Centers for Disease Control and Prevention (CDC's) "Clean Hands Count" campaign: Healthcare providers fact sheet This material was developed by The Centers for Disease Control and Prevention (CDC) (www.cdc.gov/HandHygiene)

Another initiative taken by Centers for Disease Control and Prevention (CDC) was called “My Clean Hands”. May 5th is celebrated as “World Hand Hygiene Day”, and they also commenced a campaign called "Clean Hands Count" (Figure 8-9) that will offer a new training course for healthcare providers. The "Clean Hands Count" campaign aims to improve health care provider adherence to CDC hand hygiene, address the myths and misperceptions about hand hygiene and empower patients to play a role in their care by asking or reminding healthcare providers to clean their hands [14]. CDC also has a health-care providers fact sheet to address these myths and misconceptions about hand hygiene (Figure 8):

• Alcohol-based hand sanitizer is more effective and less drying than using soap and water • Using alcohol-based hand sanitizer does NOT cause antibiotic resistance • Alcohol-based hand sanitizer does not kill Clostridium difficile, but it is still the overall recommended method for hand hygiene practice • Some healthcare providers miss certain areas when cleaning their hands • The amount of product you use matters • Glove use is not a substitute for cleaning your hands. Dirty gloves can soil your hands • On average, healthcare providers perform hand hygiene less than half of the times they should

All these measures that are taken to improve hand hygiene awareness and thus prevent the spread of infections, will only be helpful if the compliance of the health care workers to these hand hygiene practices is ensured. This can be done by setting some performance indicators to check the health care workers’ compliance and improvement in hand hygiene. The indicators include periodically checking the number of hand hygiene episodes performed by the health care worker, monitoring the volume of alcohol-based hand rub being used, monitoring the compliance of not using artificial fingernails or jewelry that may provide space for colonization of germs. To encourage the practices of hand hygiene, some rewarding policies for the health care workers should be introduced [15].

## Conclusions

There is a lack of attention given to the teaching of hand hygiene practices in the Indian medical training curriculum. Around 57% (n=298) of the respondents never received any formal training in hand hygiene throughout their course of medical undergraduate study. Only 12.2% (n=64) of the respondents had a good level of hand hygiene knowledge. To prevent the spread of infections in healthcare settings, medical students should be given proper training in hand hygiene practices right from the first year of the medical curriculum. This could be done by running workshops, holding annual seminars and making it a requisite for clinical skills assessment. 

The sample size of 523 has provided us with an insight into the awareness and knowledge of hand hygiene practices among the population of medical undergraduate students in India. However, one of the limitations of this study is that the data is skewed to more respondents from medical institutes of bigger cities like Mumbai and Navi Mumbai and does not truly represent the entire nation. Thus, further studies are required to be carried out in medical institutes located in remote areas of the country where hand hygiene compliance could be a major concern.
